# Medicine as a career choice: a comprehensive study on factors influencing Sudanese students to opt in/out medical career

**DOI:** 10.1186/s12909-023-04415-w

**Published:** 2023-06-07

**Authors:** Moez Mohammed Ibrahim Bashir, Mohmed Ahmed Fadelalla Alrayah, Mohamed Esameldeen Elsayed Mustafa, Mohammed Khalid Abdulla Maroof, Mohamed Awad Omer Hamad, Moaid Mohamedosman Ali Mohamedosman

**Affiliations:** Faculty of Medicine, University of Khartoum, ElQasr Ave, P.O. Box 103, Khartoum, Sudan

**Keywords:** Medicine, Medicine in Sudan, Medical education in Sudan

## Abstract

**Background:**

The medical profession is one of the most highly respected and desired professions among students worldwide, most likely because it provides opportunities for both a financially and socially rewarding career. However, while it has been quite established that factors such as self-interest, family pressure, friend pressure, and socioeconomic status do influence the choice of medicine among students worldwide, the exact reasons for an individual to join a medical school may actually vary worldwide. The aim of this study was to comprehensively explore factors influencing medical students to opt in/out medical careers in Sudan.

**Methods:**

An institutional based descriptive cross-sectional study was conducted at University of Khartoum in the year 2022 with a random sample of 330 students that was obtained from the medical students at the The University of Khartoum, Faculty of Medicine using stratified random sampling.

**Results:**

Self-interest was the most common factor influencing opting in (choosing) medical profession (70.6%) (n = 233), followed by getting a very high score in high-school that qualifies into the faculty (55.5%) (n = 183). Regarding the factors affecting medical students’ choices, parental pressure was the main factor (37.0%) (n = 122), followed by other relatives’ pressure (12.4%) (n = 41), and 4.2% (n = 14) chose Peer pressure. 59.7% (n = 197) of the participants stated that they were not affected by any of these factors. Most of the participants felt that the general perception of the medical profession by society is that it is prestigious and has good career opportunities, only 5.8% (n = 19) believed that it is “Not appreciated at all” by the society. A statistically significant association was found between the type of admission & parent pressure (p value 0.01). out of 330 participants, (56.1%) (n = 185) have opted out i.e. lost their interest or regretted their choice of medical career. Academic difficulties was the most common factor causing students to opt-out of the medical career (37%) (n = 122) followed by Multiple suspensions of education (35.2%) (n = 116), Current political & security conflicts in Sudan (29.7%) (n = 98), Poor quality of education (24.8%). The proportion of students having regrets for the medical profession was significantly higher among females. Over one third of the participants reported having depressive symptoms more than half days of the week. No statically significant correlation was established between the academic level and having these depressive symptoms and no statistically significant correlation was established between the decision to opt-out and the academic level (class) of the individuals (P = 0.105).

**Conclusions:**

Over half of Sudanese medical students at the University of Khartoum have already lost their interest or regretted their choice of medical career choice. Whether these future doctor chose to drop out or continue their path in the medical career suggests that they are more prone to serious hardships in their future careers. A careful comprehensive approach should further explore and try to offer solutions for problems like “Academic difficulties”, “multiple suspension of education”, and “poor quality of education” for they were the most common factors that caused medical students to opt out of the medical career.

## Background

The medical profession is one of the most highly respected and desired professions among students worldwide, most likely because it provides opportunities for both a financially and socially rewarding career. However, while it has been quite established that factors such as self-interest, family pressure, friend pressure, and socioeconomic status do influence the choice of medicine among students worldwide [1] [2], the exact reasons for an individual to join a medical school may vary between countries; In Ethiopia, the most common reason given was “interest in the field of life saving”, followed by"better income” and “social prestige.“[3], in the United Kingdom, the most common reported reasons were: “being good at science subjects”, “wanting a good interesting career”, and “always having wanted to“[4]. On the other hand, while most studies of medical education have primarily focused on illuminating the mechanisms responsible for student attrition around the world, other research on medical education tried to explore factors that drive medical students to lose their interest from the medical school and medical career as a whole; quality of education, academic factors, financial causes, and health-related reasons were all factors that were found to influence students’ choices to drop out of medical school. Furthermore studies suggest that a significant percentage of graduate and undergraduate medical students experience high rates of depression, burnout, and suicidal ideation at the same time, that premeds who experience more depressive symptoms and higher levels of burnout are more likely to drop out of their schools. This negative relationship between depressive symptoms and medical career interest was found to be more pronounced in premedical women than in men [5] [6] [7] [8] [9] [10] [11]. Despite the importance of this area, and up to our knowledge, no similar national study has been conducted to explore factors influencing medical career interest among students and factors influencing medical career choice regret. The General objective of this study is comprehensively explore factors influencing Sudanese medical students to opt in/out medical careers. By opting in a medical career we mean: having an active interest in engaging in a medical career, on the other hand by opting out we refer to losing this interest and regretting this initial choice. Researching factors influencing medical career interest aids in discovering factors that may drive students into medical careers against their self-interest and thus developing structured programs to prevent such unwanted phenomena. Studying factors that lead to medical career choice regret, such as depression and burnout, on the other hand, assists colleges and universities in implementing programs aimed at protecting the mental health of those premedical students.

## Methods

### Study design

An institutional based descriptive cross-sectional study was conducted at University of Khartoum in the year 2022.

### Study area

At the Faculty of Medicine of the University of Khartoum, the study was conducted virtually. The faculty of Medicine at the University of Khartoum is Sudan’s oldest medical institution, which was established in 1924. Additionally, it is the first medical school in Sudan that received accreditation by the World Federation of Medical Education.

### Study population

The college has a total of 2335 undergraduate students registered at the time of study during the academic year 2021–2022. In terms of inclusion criteria, all undergraduate students at the University of Khartoum – Faculty of Medicine in Khartoum in the first (Batch 98), second (Batch 97 and Batch 96), third (Batch 95), fourth (Batch 94), fifth (Batch 93), and sixth (Batch 92) academic years were included in the study, and no students were excluded.

### Sample size and sampling

A random sample of 330 students was obtained from the medical students at the The University of Khartoum, Faculty of Medicine using stratified random sampling. Sample size (n) was calculated according to the formula: n = [z2 * p * (1 - p) / e2] / [1 + (z2 * p * (1 - p) / (e2 * N))] Where: z = 1.96 for a confidence level (α) of 95%, p = proportion (expressed as a decimal), N = population size, e = margin of error.

$${\rm{z}}\,{\rm{ = }}\,{\rm{1}}{\rm{.96,}}\,{\rm{p}}\,{\rm{ = }}\,{\rm{0}}{\rm{.5,}}\,{\rm{N}}\,{\rm{ = }}\,{\rm{2335,}}\,{\rm{e}}\,{\rm{ = }}\,{\rm{0}}{\rm{.05}}$$

$$\begin{array}{l}{\rm{n}}\,{\rm{ = }}\,\left[ {{\rm{1}}{\rm{.962}}\,{\rm{*}}\,{\rm{0}}{\rm{.5}}\,{\rm{*}}\,\left( {{\rm{1}}\,{\rm{ - }}\,{\rm{0}}{\rm{.5}}} \right)\,{\rm{/}}\,{\rm{0}}{\rm{.052}}} \right]\,{\rm{/}}\\\,\left[ {{\rm{1}}\,{\rm{ + }}\,\left( {{\rm{1}}{\rm{.962}}\,{\rm{*}}\,{\rm{0}}{\rm{.5}}\,{\rm{*}}\,\left( {{\rm{1}}\,{\rm{ - }}\,{\rm{0}}{\rm{.5}}} \right)\,{\rm{/}}\,\left( {{\rm{0}}{\rm{.052}}\,{\rm{*}}\,{\rm{2335}}} \right)} \right)} \right]\end{array}$$

$${\rm{n}}\,{\rm{ = }}\,{\rm{384}}{\rm{.16}}\,{\rm{/}}\,{\rm{1}}{\rm{.1645}}\,{\rm{ = }}\,{\rm{329}}{\rm{.886}}$$

$${\rm{n}} \approx \,330$$

The sample size (with finite population correction) is equal to 330.

The study population was subdivided into 7 groups according to their academic classes, and a list containing all students in each academic class was obtained. Since the study sample size was 330, a proportionate random sample from each group was selected using the online sample randomizer at https://www.randomizer.org.

The calculated sample was further divided into 46, 44, 49, 51, 47, 45, and 48 for batches 92, 93, 94, 95, 96, 97 and 98 respectively.

### Data collection methods and tools

The data were gathered using a novel online self-administered closed-ended questionnaire that was structured by the authors in accordance with the study’s goals and objectives. Lists of all students who are currently registered at the Faculty of Medicine, University of Khartoum were obtained from the faculty administration; afterwards, each person was contacted independently, directly through their own social media accounts. This method was successful, resulting in a 100% response rate.

The questionnaire contained 19 questions divided into 3 sections as follow:

#### Section 1

Contains nine questions related to the sociodemographic characteristics of the participants including: gender, age, academic level (batch), type of Admission, marital Status, place of residence (before joining the university), place of current residence (during university), weekly expenditure, and main source of income.

#### Section 2

Contains 5 questions related to factors influencing opting in/out : 3 questions investigating the factors influencing opting in including; “Why did you choose the medical profession?”, and whether their choices were “affected by parental pressure, relatives’ pressure, peer pressure or were not affected by either of these factors, and last, the participants were asked about what they think about the “general perception of the medical profession by the society?”. The other 2 questions for opt-out included directly assessing whether or not the students have lost their interest or regretted their choice of medical profession and what factors might have caused them to lose their interest or regret their choice of medical career.

#### Section 3

Contains two questions that were meant to screen for depressive symptoms among research participants, the participants were asked how often have you been bothered by “Feeling down, depressed, or hopeless?” and “having Little interest or pleasure in doing things? (Anhedonia)” over the last two weeks. The options for these questions ranged from (Never - A few times per year - Once a month - A few times per month - Once a week - A few times per week - Every day).

### Data management and statistical analysis

Data was entered and encoded then analyzed by the computer programs -by using SPSS software. The collected data was analyzed through descriptive analysis and using the chi square and t-tests. P value < 0.05 was considered statistically significant. Data were displayed and presented in the forms of tables and charts.

## Results

### Demographic characteristics

330 students responded to the questionnaire out of 330 with an overall response rate of 100%. The mean age was 23.745 years (SD = 2.1583). Of the 330 participants, 220 (66.7%) were females, while 110 (33.3%) were males. Most participants live with their families or relatives 239 (72.4%), while 81 (24.5%) live in the University dormitory. The remainder of the demographic characteristics are illustrated in Table 1.

**Table 1:** Demographic Characteristics of Participants. Shows the age, sex, marital status, expenditure, main source of income, place of residence and type of admission

Demographic Characteristics of Participants		
Variable	Total group (n = 330)	
	N	%
**Sex**		
Male	110	33.3
Female	220	66.7
**Marital status**		
Single	320	97.0
Married	9	2.7
Widowed	1	0.3
**Type of admission (to the university)**		
General Admission	269	81.5
Private Admission	61	18.5
**Place of residence (before joining the university)**		
Urban area	291	88.2
Rural area	39	11.8
**Current place of residence during study at the university))**		
With their families or relatives	239	72.5
University dormitory	81	24.5
Other	10	3%
**Expenditure (per week)**		
Less than 10,000 SDG	97	29.4
10,000–20,000 SDG	168	50.9
More than 20,000 SDG	65	19.7
**Main source of income**:		
Parents/guardian/other relative	321	97.3
Own earnings	9	2.7
	Mean	SD
**Age (years)**	23.745	2.1583

### Opting-in & opting-out

**Fig. 1:**
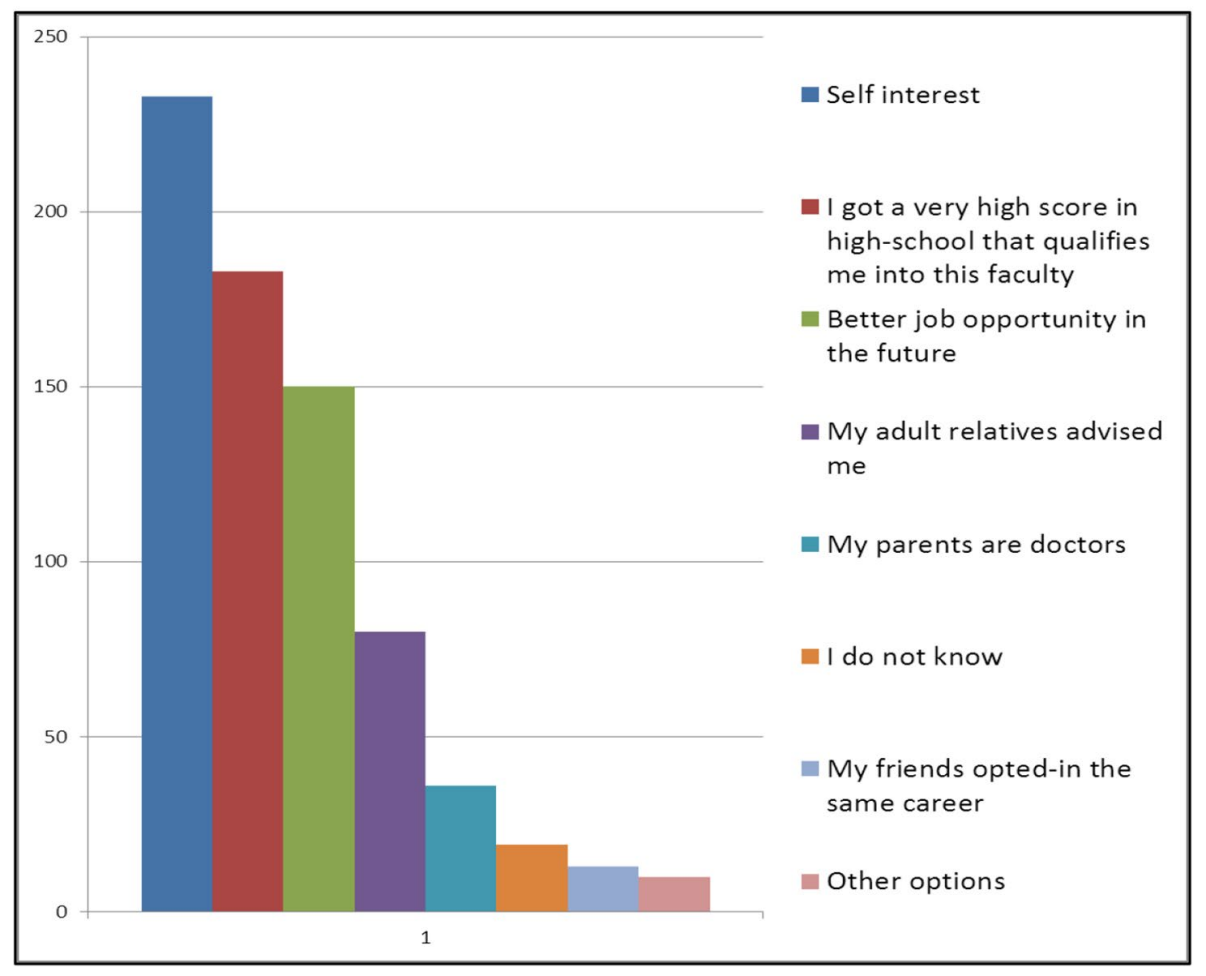
Answers for “Why did you choose the medical profession?”. Shows the reported answers provided by the participants for the question “Why did you choose medicine?”

Self-interest was the most common factor influencing opting in (choosing) medical profession (70.6%) (n = 233), followed by getting a very high score in high-school that qualifies into the faculty (55.5%) (n = 183). Better job opportunities in the future was the third factor for (45.5%) (n = 150) for our subjects. While (24.2%) (n = 80) of the participants chose “My adult relatives advised me”, 10.9% (n = 36) chose “My parents are doctors”, 5.8% (n = 19) chose “I do not know”! 3.9% (n = 13) chose “My friends opted-in the same career”. Also 3% (n = 10) of our subjects mentioned other options. Figure 1.

**Fig. 2:**
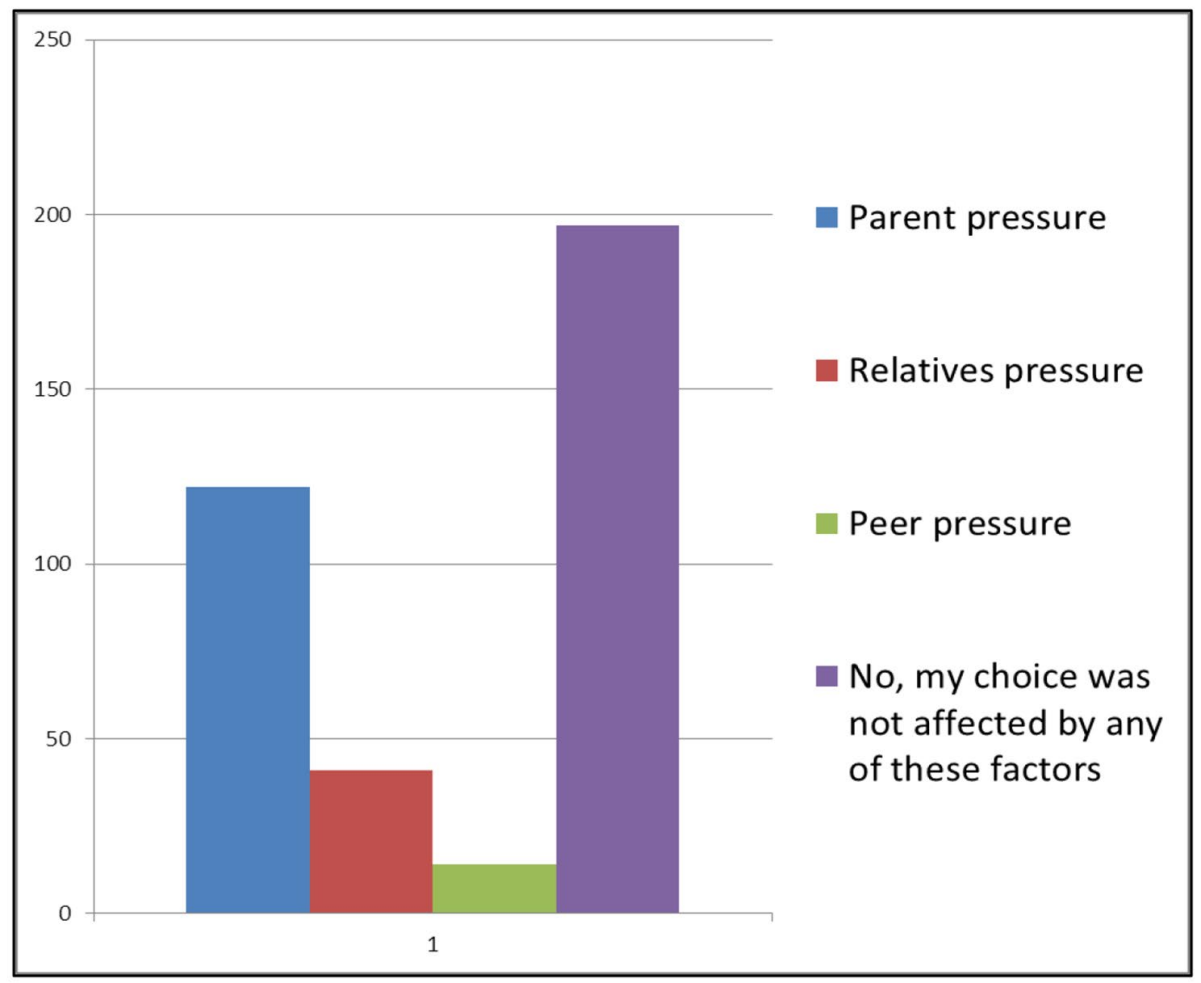
Factors influencing opting in. Shows answers to whether the participants felt that their choices were influenced by parental pressure, relatives’ pressure, peer pressure or were not influenced by either of these factors

Regarding the factors affecting medical students’ choices, parental pressure was the main factor (37.0%) (n = 122), followed by other relatives’ pressure (12.4%) (n = 41), and 4.2% (n = 14) chose Peer pressure. 59.7% (n = 197) of the participants stated that they were not affected by any of these factors. Figure 2.

**Fig. 3:**
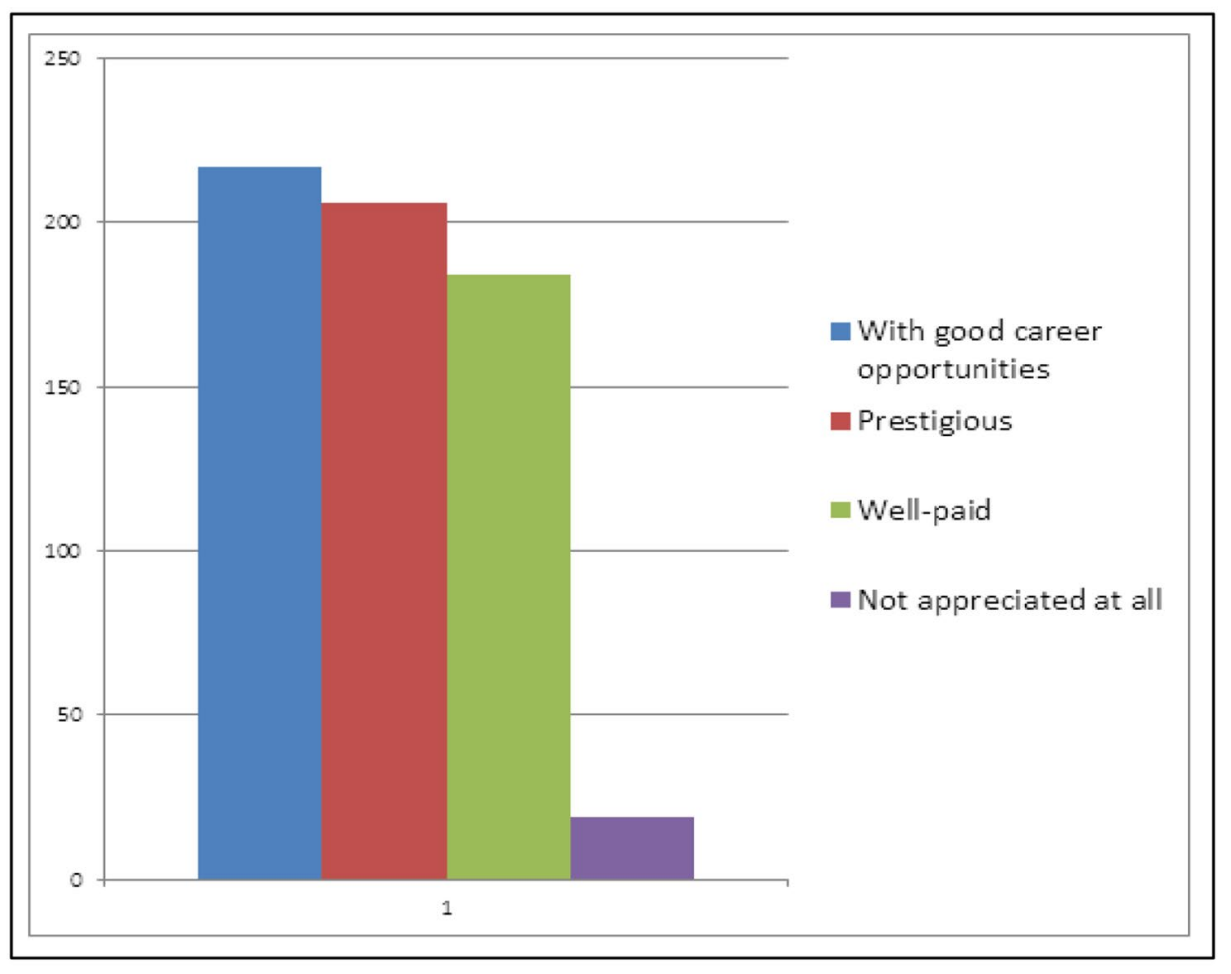
General Perception of Medical Profession by Society. Shows how medical students think the society perceive the medical profession

Regarding the general perception of the medical profession by society, most of our participants (65.8%) (n = 217) chose “With good career opportunities”, 62.4% (n = 206) chose “Prestigious”, 55.8% (n = 184) chose “Well-paid”, while 5.8% (n = 19) believed that it is “Not appreciated at all”. Figure 3.

A statistically significant association was found between the type of admission & parent pressure: Pearson correlation: 0.153 (2-tailed) (p value 0.01).

**Fig. 4:**
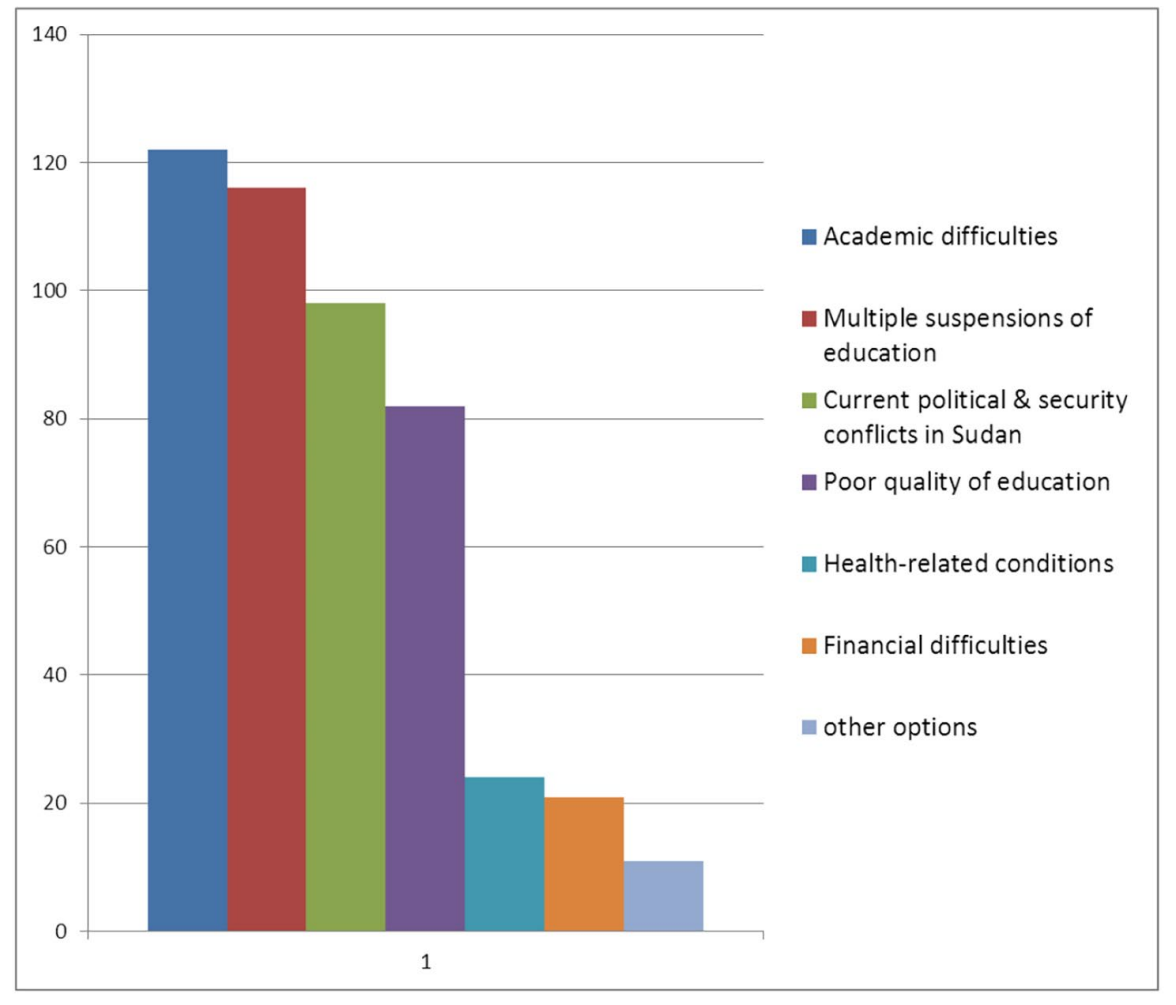
Reasons for opting out. Shows the reasons that lead our study participants to regret their choice (opt out) of a medical career

Out of 330 participants, (56.1%) (n = 185) have opted out i.e.lost their interest or regretted their choice of medical career. Regarding the factors causing opting out, Academic difficulties was the most common factor (37%) (n = 122) followed by Multiple suspensions of education (35.2%) (n = 116), Current political & security conflicts in Sudan (29.7%) (n = 98), Poor quality of education “i.e teacher and teaching method, the educational content, the learning environment and university management.” (24.8%) (n = 82), health-related conditions (7.3%) (n = 24), Financial difficulties (6.4%) (n = 21). Also (3.3%) (n = 11) mentioned other options. Figure 4.

The proportion of students having regrets for the medical profession was significantly higher among females. Pearson (0.138) (p = 0.012) Table 2.

**Table 2:** Opt out rates, causes of opt out and factors influencing opt out. Shows the percentage of career choice regret among medical students and the factors influencing this decision, 56.1% regretted their choice (opted out) of medical career. Reasons for opting out included: Academic difficulties, Financial Difficulties, Health related Reasons, Current political & security conflicts in Sudan, Poor Quality of Education, Multiple suspension of Education and Others

Variable	Total group (n = 330)		Gender			
			Males		Females	
	N	%	N	%	N	%
**Regret/Opt-out**						
Yes	185	56.1	51	46.4	134	60.9
No	145	43.9	59	53.6	86	39.1
**Reasons for opting out**:						
Academic difficulties	122	37	28	25.5	94	42.7
Financial difficulties	21	7.3	9	8.2	12	5.5
Health-related reasons “I have a medical disease or other health-related conditions,e.g: Depression, Anxiety, Drug/Alcohol addiction etc.”	24	0.3	4	3.6	20	9.1
Current political & security conflicts in Sudan	98	29.7	30	27.5	68	30.9
Poor quality of education-	82	24.8	25	22.9	57	25.9
Multiple suspensions of education “i.e the University closes down a lot!”	116	35.2	34	31.2	82	37.3
Others	11	3.3	2	1.8	9	4.1
**Screening for depressive symptoms**:						
Little interest or pleasure in doing things? (Anhedonia)						
At least More than half of the days	116	35.2	36	32.7	80	36.3
Nearly everyday	54	16.4	16	14.5	38	17.2
Feeling down, depressed, or hopeless?						
At least More than half of the days	113	34.3	34	30.9	79	35.9
Nearly everyday	50	15.2	15	13.6	35	15.9

In light of the data collected, it is evident that while there is a certain level of variance in the opt-out rates among the participants, no statistically significant correlation was established between the decision to opt-out and the academic level (class) of the individuals (P = 0.105).

### Screening for depressive symptoms

Among the 330 entries 35.2% (n = 116) reported having “little interest or pleasure in doing things” (anhedonia) at least more than half the days over the last two weeks. 16.4% (n = 54) reported having “little interest or pleasure in doing things” (anhedonia) nearly everyday over the last two weeks. 34.3% (n = 113) reported feeling “down, depressed or hopeless” at least more than half the days over the last two weeks. 15.2% (n = 50) reported feeling “down, depressed or hopeless” nearly everyday over the last two weeks. Between different academic level, fourth-year students (Batch 94) were the most common to report reported having “little interest or pleasure in doing things” (anhedonia) with a percentage 46.9% (n = 23) reported having the symptoms at least more than half the days over the last two weeks and 26.5% (n = 13) of students reported having the symptoms nearly everyday over the last two weeks, students of Batch 94 were also the most common students report feeling “down, depressed or hopeless” with a percentage of 42.8% (n = 21) reported having the symptoms at least more than half the days over the last two weeks and 22.8% (n = 11) reported reported having them nearly everyday over the last two weeks. However no statically significant correlation was established between the academic level and having these depressive symptoms (P = 0.1). A significant association was found between Opting out and having “little interest or pleasure in doing things” (anhedonia) over the last two weeks (p = 0.0001). A significant association was found between opting out and feeling “down, depressed or hopeless” over the last two weeks (p = 0.0001). Table 3.

**Table 3:** Regret and Depressive symptoms according to academic level (Batch). Shows the percentage of regret/opting out and depressive symptoms rates in each of the academic levels

Academic Level (Batch)	Regret/opt out		Screening for depressive symptoms							
			Little interest or pleasure in doing things? (Anhedonia)				Feeling down, depressed, or hopeless?			
			At least More than half of the days		Nearly everyday		At least More than half of the days		Nearly everyday	
	N	%	N	%	N	%	N	%	N	%
92	23	50	11	23.9	5	10.8	8	17.3	5	10.8
93	27	61.4	12	27.2	3	6.8	13	29.5	4	4.3
94	32	65.3	23	46.9	13	26.5	21	42.8	11	22.4
95	29	56.9	23	45.1	11	21.5	18	35.3	9	17.6
96	29	61.7	14	29.8	5	10.6	18	38.3	8	17
97	27	60	17	37.8	9	20	17	37.8	6	13.3
98	18	37.5	16	32.7	8	16.3	18	36.7	7	14.3

## Discussion

This study was to conducted to identify factors influencing Sudanese medical students to opt in/out medical careers. This study included 330 students two third of which were females. Most (88.2%) of the participants were urban residents. The type of admission 269(81.5%) were classified as General admission / and 61 (18.5%) as Private admission, this finding is consistent with the fact that the general admission allocated seats in the faculty represent about 80% of the total number of seats when compared to the seats allocated for private admission.

Our study found that the most common factor for opting in a medical profession was self interest (70.6%). Choosing “self interest” as a factor for opting in reflects intrinsic motivation and hence having the vast majority of participants select this choice is quite reassuring. It is fair to say that this finding is relatively consistent with some studies in literature; self interest was the most common factor (86.4%) in a study conducted in Saudi Arabia [10]. A study in India and a Finnish national study in which self interest was also a motive for 82.6% and 77–82% of the studies’ participants respectively. [1] [12].

The second most common factors for opting-in found in our study was getting a very high score in high-school that qualifies into the faculty; logically, most of students who opt in a medical career and enrolled in the faculty of medicine actually would have already gotten a very high score that qualifies them into the faculty of medicine, this is why it’s understandable to have the majority of the participants choosing this option as one of the factors that caused them to opt in a medical profession. However, what is really interesting is the fact that we found 4.6% (n = 15) of the participants stating that they opted in the medical profession only because they got a very high score in high-school that qualified them into the faculty”!

Also interestingly 3% (n = 10) of our subjects decided to write their own answers to our question “*Why did you choose the medical profession?*” under the section “*other options*”. Upon analyzing these answers, we found that they are markedly diverse, ranging from answers that show substantial interest like :“*I love medicine*” and “*I want to help people and develop the health status in my country*” to ambiguous and uncertain answers like :“*there are no other choices*” and “*I did not see any better option*” to answers that completely indicate that the student did not even choose to opt in the medical career by themselves like :“*I did not choose it, my family did*” And “my parents forced me”!

Unlike other studies investigating factors that influence opting in medical career [10], [13], [14], 18, [1], in our study, we allocated a separate question to explore factors that influence opting in in addition to the primary factors that caused the participants to opt in a medical career; this may partly explain the increased percentage for opt-in influences that may be noticed in our study when compared to other studies, for instance, we found that parental pressure (37.0%) was the most common factor that influenced the choice of opting in medical career, followed by relatives pressure (12.4%), and peer pressure being the least to influence opting in only (4.2%).

We think that the percentage of parents’ pressure in our study (37%) is high when compared to other similar studies where we have seen percentages as little as 0.66%, 7.3% and 11.6% [1] [2] [10]. This is a very interesting finding and may reflect the highly prestigious and valued view of the community towards the medical profession in our country, which might have influenced most parents to make them want their son and daughters to become future doctors. Interestingly, we found that more than half of the private admission participants mentioned that they have been influenced by parents pressure with a statistically significant association between the type of admission & parent pressure: Pearson correlation: 0.153 (2-tailed) (p value 0.01) When compared to general admission participants; This might indicate that high percentage of private admission students are forced by their parents to pursue a medical profession. This can be explained by the fact that students logically face more pressure to achieve their family desires, the more their families spend on their education, bearing in mind that the vast majority of our population depend primarily on parents/guardians as their source of income.

When we asked our participants about their general perception of the medical profession by society, the majority regarded it with good career opportunities (65.8%), Prestigious (62.4%), and Well-paid (55.8%). From our point of view, these results reflect and confirm the positive society impression towards them. Despite that we found 5.8% of the population believe that the medical profession is not appreciated at all.

Regarding opting out, we believe that the study has uncovered some interesting and quite alarming findings, having over half of the medical students at the University of Khartoum (56.1%) losing their interest or regretting their choice of medical career. Up to our knowledge, this is one of the highest percentages ever to be reported on such a subject, in Telangana, India, for instance, a study mentioned that “It was shocking to see almost 40% of the students were having regret feeling for choosing medical profession.” [1], another study among medical students in Kazakhstan found that 33.0% of the responders reported that they regretted their previous choice of the profession. [2]

Overall, we found this level of regret is even higher than the level of career choice regret among healthcare professionals and potential healthcare professionals during the COVID-19 pandemic (40.9%) which indeed was a highly stressful and catastrophic situation. [12] The past few years could be considered quite stressful in Sudan, specially for medical students at the University of Khartoum, as since December 2018, most students have studied not more than a total of 5 semesters only, due to the multiple suspensions of in-campus education for multiple reasons over the past years, though It was surprising that “Academic Difficulties” and not “Multiple suspension of education”, this can be drawn to the relatively higher academic workload in the university when compared to lower ranking universities in Sudan. Moreover, the fact that 24.8% of students stated that “poor quality of education” is one of the reason behind their choice regret, also does draws a lot of question marks, since -up until now- University of Khartoum, Faculty of Medicine is the highest ranking university in Sudan, and having nearly one quarter of students’ not only labeling it as having “poor quality of education” but also regretting their choices of joining the medical field because of this poor quality is sure a quite worrisome fact. And since by “Quality of Education” we referred to teacher and teaching method, the educational content, the learning environment and university management, studies investigating the these quality of these factors at the University of Khartoum and Sudanese medical schools as a whole is necessary to objectively examine the quality of education in these facilities.

Overall, this situation is quite similar to a nearby country, Ethiopia, in which 35% of students of medical school at Addis Ababa University, the best in the country, felt the standard of medical education was below their expectation [3]. From another view the recent changes in medical education following the pandemic may have a role in these results as online teaching alone was labeled not preferred by majority of students in Hamdard institute India [15], in another study reasons for regret feeling for choosing medicine as career included: “Lifelong reading 36%”, “Feeling Stress 19%” and “Frequent exams 1%” [1] which are still close to our findings, but it’s still difficult to compare the results of our study with other studies, first due to the considerable heterogeneity of the definition of dissatisfaction or regret, and second due to the wide difference in the sociodemographic and geopolitical backgrounds between the two countries, this highlights the need for cross-national comprehensive studies that fully explore this subject.

We believe that there are some further hidden factors that attribute to the regret of choice among medical students, this is very evident due to the fact that a statistically significant correlation was found between opting out and having “little interest or pleasure in doing things” (anhedonia) over the last two weeks and also between opting out and feeling “down, depressed or hopeless” over the last two weeks, these two findings highly suggest that symptoms of depression can very well be one of these aforementioned hidden factors and it is fair to say you can never tell which one comes first. Self-interest was the main reason for opting in in our research, and it’s only logical that students who have lost their interest in doing things that were once enjoyable lose their interest in their studies and regret their choice.

Career choice regret is an established risk factor for burnout [16] But we also believe the other way around is also possible, it’s logical to think that burnout can as well influence medical students into regretting their choice of getting into the medical field [17][18][19][20][21] Although this study suggested a correlation between burnout and career choice regret among medical students, the need for testing a full causal relationship between burnout and career choice regret by further research still exists.

### Limitations

Being conducted among the medical students at the University of Khartoum the generalizability of this study to the broader Sudanese medical students population is relatively limited and further studies should be conducted to fully inspect the whole Sudanese medical students community. Generalizing the study to the global community of medical students is also limited due to cultural and contextual factors that are found uniquely in Sudan. Another notable limitation was due to the study design itself, as since this was a cross-sectional study assessing changes in factors influencing opting in/out over time was limited and hence further longitudinal studies should be conducted to assess changes in factors over a longer period of time.

## Conclusions

Over half of Sudanese medical students at the University of Khartoum have already lost their interest or regretted their choice of medical career choice. Whether these future doctor chose to drop out or continue their path in the medical career suggests that they are more prone to serious hardships in their future careers. A careful comprehensive approach should further explore and try to offer solutions for problems like “Academic difficulties”, “multiple suspension of education”, and “poor quality of education” for they were the most common factors that caused medical students to opt out of the medical career.

## Data Availability

The datasets used and analyzed during the current study are available upon reasonable request from the corresponding author.
